# Editorial Comment: Step-by-step Laparoscopic Vesiculectomy for Hemospermia

**DOI:** 10.1590/S1677-5538.IBJU.2016.0127.1

**Published:** 2017

**Authors:** Tariq S. Hakky

**Affiliations:** 1 Department of Urology, University of Southern Florida, Moffitt Cancer Center, USA

## Abstract

In this video Mello et al. (1), the authors highlight the clinical merit of step-step laproscopic vesiculectomy for hemospermia. The authors adopt a robotic minimally invasive surgery to the realm of seminal vesiculectomy, which was first highlighted by Kavoussi et al. in 1993 (2). It depicts an easy step-by step approach and nicely demonstrates how to mange the vascular pedicle. The present video highlights that that this be accomplished to address an underlying clinical manifestation requiring surgical resection. In their series Mello et al. (1) pathological analysis showed amyloidosis, and transitional epithelium without atypia. With the advantage of combined 3D vision and wristed instrumentation, robotic excision of the seminal vesicles is feasible, safe and regarded as a natural continuity of conventional laparoscopy.

*Tariq S. Hakky, MD* *Department of Urology* *University of Southern Florida, USA* *Moffitt Cancer Center, USA* *E-mail: tariq7@gmail.com*
